# A Rare Case of Possible Iron Overload and B12 Deficiency in Autoimmune Polyglandular Syndrome Type 3B

**DOI:** 10.7759/cureus.114930

**Published:** 2026-08-21

**Authors:** Katie Kyan, Rosie Kumar, Debra Craig

**Affiliations:** 1 Internal Medicine, California University of Science and Medicine, Colton, USA; 2 Internal Medicine, Arrowhead Regional Medical Center, Colton, USA

**Keywords:** aps3b, autoimmune hypothyroidism, autoimmune polyglandular syndrome type 3, iron overload, pernicious anemia, vitamin b12 deficiency anemia

## Abstract

Autoimmune polyglandular syndromes (APS) are a rare group of disorders characterized by the dysfunction of multiple endocrine and non-endocrine organs due to autoimmune destruction. APS type 3B (APS3B) is a subtype characterized by the coexistence of autoimmune thyroiditis and pernicious anemia, more commonly observed among middle-aged females. Here, we describe a unique case, workup, and diagnosis of APS3B in a 58-year-old Hispanic male who presented with an atypical presentation of APS3B complicated by metformin use and possible transfusional iron overload. Workup revealed positive intrinsic factor antibodies and elevated homocysteine and methylmalonic acid levels, confirming pernicious anemia. Thyroid studies demonstrated markedly positive anti-thyroid peroxidase antibodies and elevated thyroid-stimulating hormone, consistent with autoimmune thyroiditis. He was treated with blood transfusions, initiation of intramuscular vitamin B12 injections, and discontinuation of metformin. This case highlights how APS may be overlooked in patients with multiple chronic comorbid conditions and should be considered in those presenting with several endocrine abnormalities.

## Introduction

Autoimmune polyglandular syndromes (APS) are a rare group of disorders characterized by the presence of at least two autoimmune-induced endocrine diseases [1]. The three main types are recognized as APS1, APS2, and APS3. Clinical diagnosis of APS1 requires two of the following: chronic mucocutaneous candidiasis, hypoparathyroidism, and autoimmune adrenal insufficiency, often referred to as Addison’s disease [2]. APS2 is characterized by autoimmune adrenal insufficiency with autoimmune thyroid disease and/or type I diabetes mellitus (T1DM) [3]. In contrast, APS3 is diagnosed when there is autoimmune thyroid disease plus at least one other autoimmune condition, excluding adrenal insufficiency, hypoparathyroidism, and chronic candidiasis [4]. 

Autoimmune diseases associated with APS3 include T1DM, vitiligo, myasthenia gravis, autoimmune hepatitis, pernicious anemia, and Sjögren's syndrome [4]. Although the prevalence of APS3 is estimated at one in 20,000 individuals in the United States, it remains the most common type of APS and occurs more frequently in middle-aged females [5]. One study suggested that approximately 52% of patients with autoimmune thyroid disease may have findings consistent with APS3 [6]. It can further be classified into three subtypes: APS3A, autoimmune thyroiditis with immune-mediated diabetes mellitus; APS3B, autoimmune thyroiditis with pernicious anemia; and APS3C, autoimmune thyroiditis with vitiligo and/or alopecia and/or other organ-specific autoimmune disease [6]. This case report focuses on APS3B, highlighting an unusual presentation of chronic vitamin B12 deficiency with macrocytic anemia and possible transfusional iron overload. 

This article was previously presented as a meeting abstract at the 2026 Society of Hospital Medicine Converge Meeting on March 31, 2026.

## Case presentation

Initial presentation

A 58-year-old Hispanic male with a past medical history of chronic vitamin B12-deficiency macrocytic anemia, recurrent blood transfusions, chronic myositis, type II diabetes mellitus (T2DM) on metformin, hypothyroidism, and peripheral neuropathy presented to the emergency department with generalized fatigue, weakness, and dyspnea on exertion that had been worsening for about one and a half months. He also reported dizziness, loss of appetite, nausea, and generalized swelling of his hands, feet, and face, which was more pronounced in the mornings. 

The patient states that he has experienced these symptoms in the past with noted improvement after eating food and receiving blood transfusions. He was first diagnosed with chronic macrocytic anemia about 15 to 20 years ago and has needed blood transfusions approximately every six months since then. His daughter, present at the bedside, reported that he was previously scheduled with his primary care provider (PCP) for B12 intramuscular injections but has not followed up for over a year. The patient was seen at our facility about two years ago for similar symptoms and was transfused with two units of packed red blood cells (PRBCs) on admission, with improvement of symptoms. He was advised to follow up with hematology after discharge for a possible bone marrow biopsy but was lost to follow-up. 

Additionally, two weeks before presentation, the patient restarted taking metformin 1,000 mg twice daily after not taking any medications for his T2DM for about six months. Other home medications included levothyroxine 125 mcg daily for hypothyroidism, gabapentin 300 mg daily for peripheral neuropathy, and vitamin B12 supplements of unknown dose for his vitamin B12-deficiency macrocytic anemia. 

In the emergency department, the patient’s vital signs were significant for tachycardia, and the physical examination was notable for facial edema, bilateral eyelid swelling, mild swelling of bilateral hands and lower legs, and pallor. The patient was given ondansetron for reported nausea and was started on oral vitamin B1 and folic acid supplements for a history of macrocytic anemia. 

Initial laboratory findings revealed macrocytic anemia with a significantly low hemoglobin of 5.6 g/dL and a mean corpuscular volume of 121 fL. Mild leukopenia was also noted. Vitamin B12 levels were markedly decreased. Iron studies demonstrated evidence of possible iron overload, with significantly elevated iron, ferritin, and transferrin saturation levels. The comprehensive metabolic panel was remarkable for elevated blood glucose and liver enzymes. A detailed summary of these laboratory findings is presented in Table 1. Additional testing, including troponin, folate levels, urinalysis, urine drug screen, and infectious screenings for hepatitis C and syphilis, were unremarkable. Electrocardiogram demonstrated sinus tachycardia without other abnormalities. The patient was transfused with two units of PRBCs and continued on his home medications of levothyroxine and gabapentin while in the emergency department. 

**Table 1:** Pertinent laboratory results at presentation AST, aspartate aminotransferase; MCH, mean corpuscular hemoglobin; MCV, mean corpuscular volume; RBC, red blood cell; RDW, red cell distribution width; WBC, white blood cell.

Laboratory test	Measured value	Reference range
Complete blood count		
Hemoglobin (g/dL)	5.6	13.0-17.0
Hematocrit (%)	16	41-53
RBC count (×10⁶/µL)	1.34	4.5-5.9
WBC count (×10^3^/µL)	3.8	4.5-11.1
MCV (fL)	121	80.0-100.0
MCH (pg)	41.8	26.0-32.0
RDW (%)	16	11.0-15.0
Comprehensive metabolic panel		
Blood glucose (mg/dL)	204	65-125
AST (U/L)	81	5-40
Total bilirubin (mg/dL)	1.7	0.0-1.2
Direct bilirubin (mg/dL)	0.8	0.0-0.4
Iron panel		
Iron (μg/dL)	193	50-160
Ferritin (ng/mL)	583	250-450
Transferrin saturation (%)	>92	20-50
Other tests		
Vitamin B12 (pg/mL)	<150.0	250-1,100

Hospital course

The patient was admitted for further workup of symptomatic macrocytic anemia in the setting of severe B12 deficiency. During his admission, he was transfused with another one unit of PRBCs with improvement of hemoglobin from 6.6 to 7.8 g/dL. Hematology was consulted and recommended starting B12 intramuscular injections of 1,000 mcg for seven days, followed by weekly for four weeks, then monthly for maintenance.

Laboratory results

Further laboratory studies revealed positive intrinsic factor antibodies and elevated homocysteine, confirming vitamin B12 deficiency secondary to pernicious anemia. Vitamin B1 levels were within normal limits, and the sickle cell screen was negative. Autoimmune workup for the patient’s chronic myositis revealed elevated aldolase and C-reactive protein, with normal creatine kinase levels. Thyroid panel was consistent with the patient's history of hypothyroidism, and glycated hemoglobin was 6.8%. Other studies to evaluate possible autoimmune conditions included parathyroid hormone (PTH) and cortisol levels, which were within normal limits throughout the admission. A summary of admission findings is presented in Table 2.

**Table 2:** Pertinent laboratory results during admission CRP, C-reactive protein; HbA1c, glycated hemoglobin; OR, odds ratio; TSH, thyroid-stimulating hormone.

Laboratory test	Measured value	Reference range
HbA1c (%)	6.8	4.3-6.1
Homocysteine (µmol/L)	55.8	<11.4
TSH (mIU/L)	36.1	0.35-5.5
Free T4 (ng/dL)	0.53	0.80-1.50
Intrinsic factor antibodies	Positive	Negative
Aldolase (U/L)	33.8
CRP (mg/dL)	1.74	<0.50
Creatinine kinase (U/L)	52	30-170

Imaging results

**Figure 1:**
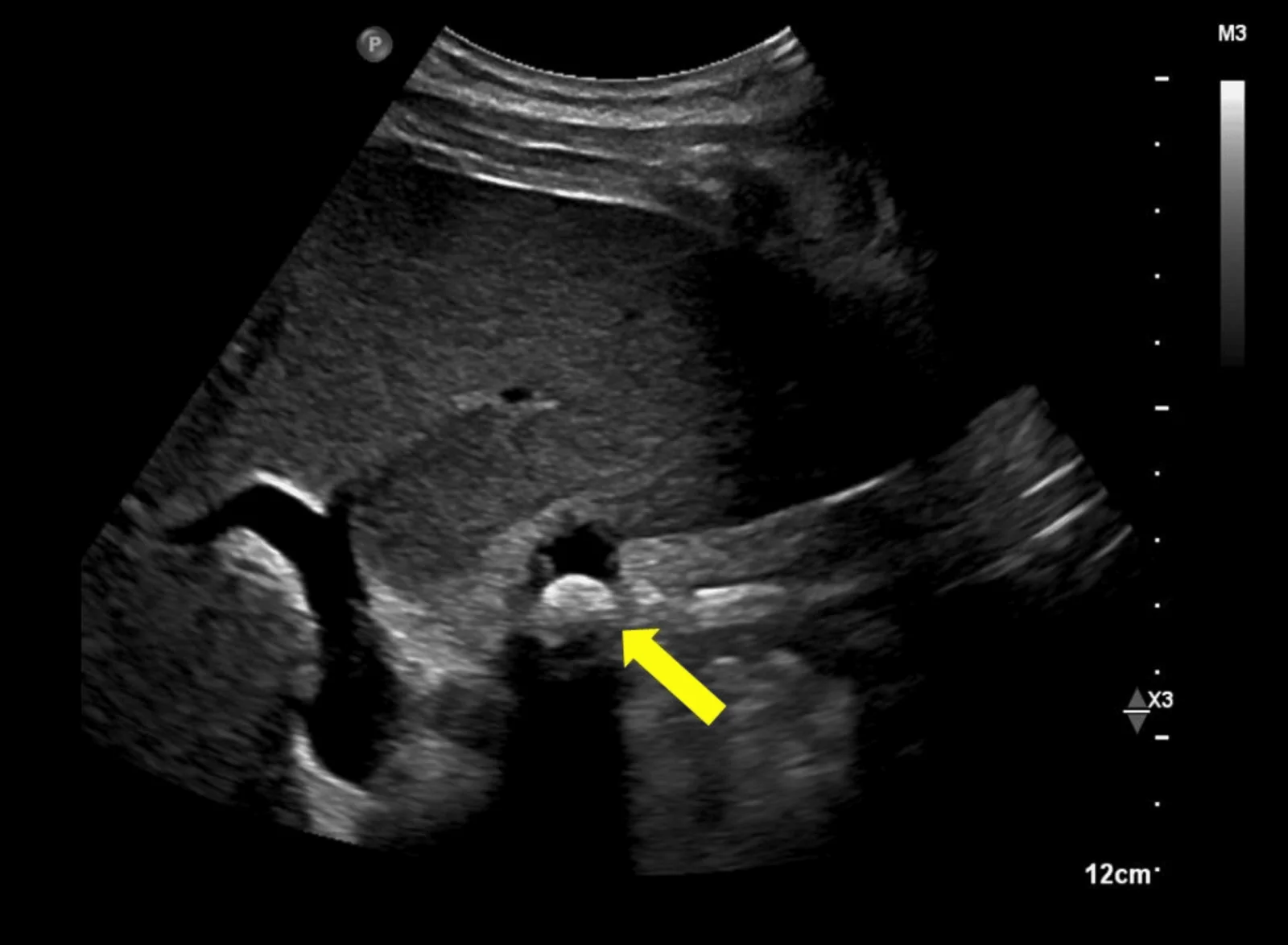
Transverse abdominal ultrasound demonstrating an echogenic gallstone (arrow) within the gallbladder, consistent with cholelithiasis.

**Figure 2:**
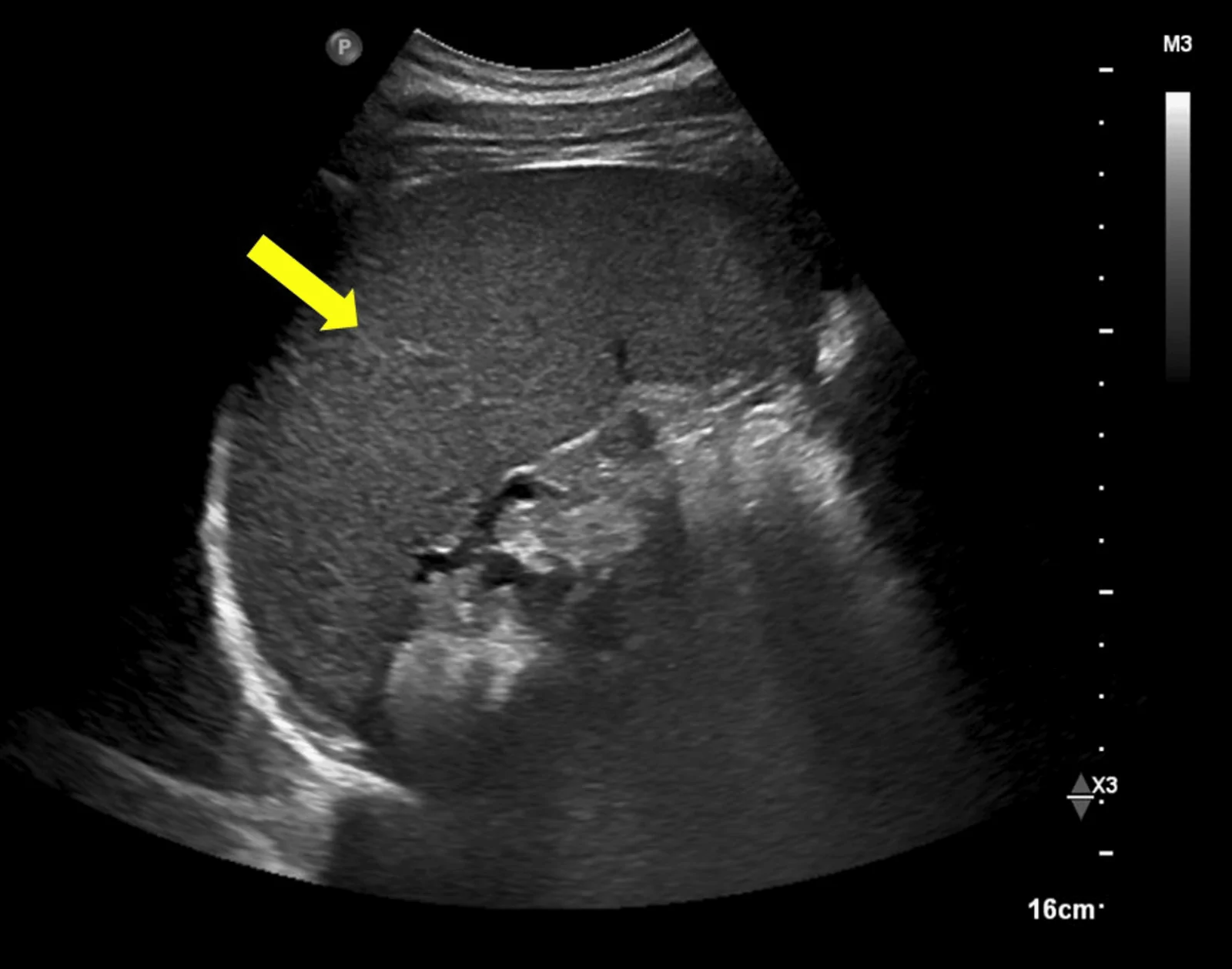
Longitudinal abdominal ultrasound demonstrating mild splenomegaly. Arrow indicates the spleen.

Abdominal ultrasound was performed to evaluate elevated liver enzymes and re-demonstrated cholelithiasis and mild splenomegaly, findings unchanged from a prior ultrasound 13 years ago (Figures 1, 2). A transthoracic echocardiogram was obtained to evaluate cardiac function, given concern for iron overload, and revealed a left ventricular ejection fraction of 60%, with normal right and left ventricular size and function.

Discharge plan

The patient’s hemoglobin stabilized at 8.2 g/dL, with clinical improvement in his symptoms, and he was discharged with oral B12 5,000 mcg supplements, a hematology referral, and instructions to follow up with hematology for 1,000 mcg intramuscular injections. He was also recommended to receive an outpatient cardiac MRI to evaluate for hemochromatosis, given that he had received multiple blood transfusions and his labs showed evidence of possible iron overload. He was discontinued on metformin, as this drug is known to impair vitamin B12 absorption. No alternative diabetes medications were initiated at this time due to his A1c being within an acceptable range. 

Post-discharge results

Pending laboratory results returned after discharge and were notable for a significantly elevated methylmalonic acid level, further confirming vitamin B12 deficiency secondary to pernicious anemia. Anti-thyroid peroxidase antibodies were markedly positive, consistent with autoimmune thyroiditis as the cause of the patient’s hypothyroidism. PTH-related peptide was slightly decreased, but calcium and PTH levels were normal. Testing for hereditary hemochromatosis was negative, making a genetic iron overload disorder unlikely in this patient. Sjögren's antibodies and antinuclear antibody test were both negative, making Sjögren’s syndrome and systemic lupus erythematosus unlikely as well. A summary of post-discharge laboratory results is presented in Table 3.

**Table 3:** Laboratory results after discharge ANA, antinuclear antibody; PTHrP, parathyroid hormone-related peptide; TPO, thyroid peroxidase.

Laboratory test	Measured value	Reference range
Methylmalonic acid (nmol/L)	1,678	55-335
Anti-TPO antibodies (IU/mL)	>900	<9
ANA screen	Negative	Negative
Sjögren's antibodies	Negative	Negative
PTHrP (pg/mL)	6	11-20
Hereditary hemochromatosis	Negative	Negative

## Discussion

This patient’s findings of autoimmune thyroiditis with positive thyroid peroxidase antibodies and pernicious anemia with positive intrinsic factor antibodies meet the criteria for APS3B. Although APS3 is more common among middle-aged females, this case report stands out as an atypical presentation in a 58-year-old Hispanic male [5]. 

While the patient’s elevated iron indices may be related to transfusional iron overload given his longstanding history of recurrent blood transfusions, the extent of tissue iron accumulation could not be determined during this admission. One unit of transfused blood contains about 200 to 250 mg of iron, and those who receive as few as 10 to 20 lifetime transfusions are at risk of developing iron overload [7]. This may be the case for the patient, as he reported needing transfusions about every six months for the past 15 to 20 years due to long-standing, inadequately treated chronic anemia. However, no specific interventions, such as chelation therapy, were initiated during this admission, as there was no evidence of organ damage, given an unremarkable abdominal ultrasound and transthoracic echocardiogram. In addition, chelation therapy is usually begun when serum ferritin is greater than 1,000 ng/mL, and this patient’s ferritin was only 583 ng/mL [8]. Nonetheless, his elevated iron markers and a history of recurrent transfusions underscore the importance of ongoing surveillance such as with cardiac MRI, liver MRI, and routine ferritin levels to assess for iron-related organ dysfunction [8].

Prior research on APS2 has linked abnormalities in iron metabolism to systemic inflammation and adrenal or thyroid hormone deficiency [9]. Similarly, autoimmune thyroid disease has been associated with an increased risk of hemochromatosis, with men affected by hemochromatosis having an 80-fold higher risk of thyroid dysfunction [10]. These findings may be relevant to our patient, as abnormalities in iron metabolism have been associated with thyroid dysfunction. However, further research is needed to investigate the potential role of hemochromatosis in autoimmune conditions such as APS.

Therapeutic management in this case was complicated by the patient’s reinitiation of metformin, which may have contributed to or exacerbated his pre-existing B12 deficiency, as this drug is known to impair vitamin B12 absorption in the gut, particularly in patients with underlying malabsorptive conditions such as pernicious anemia [11]. Few studies have evaluated the treatment of T2DM in patients with pernicious anemia. However, the general consensus is that in patients with a history of B12 deficiency, it is important to monitor B12 levels regularly when prescribing or continuing metformin and to consider alternative diabetes medications that do not interfere with vitamin B12 absorption. If metformin use is necessary, regular vitamin B12 supplementation should be part of the management plan, along with routine assessments of vitamin B12 and homocysteine levels [11]. In this case, the patient was placed on a pre-prandial insulin sliding scale to control his blood sugars while in the hospital, with discontinuation of metformin on discharge.

Although this patient had a known history of chronic vitamin B12 deficiency, the underlying cause of pernicious anemia had not been formally diagnosed due to limited outpatient follow-up. Before presentation, he was taking oral B12 supplements of an unknown dosage, but he likely required higher-dose therapy or intramuscular injections given his malabsorptive condition. Establishing the diagnosis of pernicious anemia during this admission clarified the need for lifelong monthly B12 injections. Although hypothyroidism can also contribute to anemia, it was unlikely to fully account for the degree of macrocytosis and anemia observed in this patient. This case illustrates how long-standing B12 deficiency can sometimes mask or delay recognition of pernicious anemia, especially when symptoms can be misattributed to other chronic conditions such as diabetes-related neuropathy.

Other future considerations include human leukocyte antigen typing, which was not conducted in this patient but could be considered, particularly for family screening, to help identify individuals at risk for autoimmune conditions such as autoimmune thyroiditis and pernicious anemia [6]. Overall, management of APS centers on addressing the individual conditions of the syndrome. This often involves hormone replacement therapy, lifelong monitoring, and regular screening for the development of additional autoimmune diseases, given the broad and evolving nature of APS. The patient was advised to follow up with his PCP for continuity of care. For patients with multiple chronic conditions and a potential autoimmune etiology, timely diagnosis, appropriate monitoring, and long-term management are essential. 

## Conclusions

Given the patient’s autoimmune thyroiditis and pernicious anemia, he meets the criteria for APS3B. This case highlights how APS3B can present with a constellation of symptoms that may appear unrelated at first glance. Symptoms can range from macrocytic anemia and neuropathy to fatigue, dyspnea, and generalized swelling. Moreover, the potential for transfusional iron overload should be considered in patients with a chronic history of anemia and recurrent blood transfusions. While these overlapping features can be misattributed to more common conditions such as diabetes or nutritional deficiencies, it is important to maintain a high index of suspicion for autoimmune syndromes in complex, multisystem presentations.
